# Resveratrol pre-treatment alleviated caerulein-induced acute pancreatitis in high-fat diet-feeding mice via suppressing the NF-κB proinflammatory signaling and improving the gut microbiota

**DOI:** 10.1186/s12906-022-03664-4

**Published:** 2022-07-16

**Authors:** Xiaoying Zhang, Guodong Yang, Yulin Chen, Zhao Mu, Haiyue Zhou, Luoyao Zhang

**Affiliations:** 1 School of Basic Medicine, North Sichuan Medical College, Nanchong, 637000 Sichuan China; 2 Department of Gastroenterology and Hepatology, Affiliated Hospital of North Sichuan Medical College, No.63, Cultural Rd., Shunqing Dist, Nanchong, 637000 Sichuan Province China; 3 North Sichuan Medical College, Nanchong, 637000 Sichuan China

**Keywords:** Resveratrol, Hyperlipidemia acute pancreatitis, Caerulein, Gut microbiota, Inflammation

## Abstract

**Background:**

hyperlipidemia acute pancreatitis (HTG-AP) is a major hidden danger affecting human health, however, whether there is a protective effect of resveratrol on HTG-AP is unclear. Therefore our study was aimed to investigate the preventive effect and the underlying mechanism of resveratrol in the HTG-AP mice model.

**Methods:**

This research was divided into two parts. In the first part, mice were adaptively fed with normal chow or HFD for 6 weeks. From the second week, resveratrol-treated mice were in intragastric administration with resveratrol (45 mg/kg/d) for 4 weeks. In the second part, the procedures were the same as the first part. After the last intragastric administration with resveratrol, all mice were intraperitoneal injections of cerulean.

**Results:**

We found resveratrol effectively inhibited pancreatic pathological injury in the HFD, AP, and HTG-AP mice. Resveratrol reduced the LPS, IL-6, TNF-α, and MCP-1 expressions in the HFD mice. Resveratrol also reduced TNF-α, MDA, and MCP-1 expressions and increased SOD and T-AOC expressions in the AP and HTG-AP mice. Furthermore, resveratrol suppressed the NF-κB pro-inflammatory signaling pathway in pancreatic tissues in the AP and HTG-AP mice. Moreover, resveratrol improved the gut microbiota in the HFD mice.

**Conclusion:**

The resveratrol pre-treatment could attenuate pancreas injury, inflammation, and oxidative stress in the HTG-AP mice, via restraining the NF-κB signaling pathway and regulating gut microbiota. Therefore, Our study proved that the resveratrol pre-treatment had a preventive effect on HTG-AP.

## Background

The annual incidence of acute pancreatitis (AP) is between 13 and 45 cases per 100,000 populations worldwide [1]. Hypertriglyceridemia (HTG) is the third most common cause of AP accounting for up to 7% of the cases [2] with gallstones accounting for 60% [3] and alcohol for 30% [4]. Whereas, HTG is the most common established cause in some specific physiological states, such as pregnancy [5]. The clinical course of HTG-AP is highly similar to other causes of AP, but HTG is the only significant clinical feature. Furthermore, HTG-AP is often accompanied by higher severity and an increased complication rate [6]. Clinical studies have reported that HTG aggravated the course of AP and aggravated the inflammatory response [7, 8]. However, the underlying mechanism is still unclear. Therefore, a novel effective and risk-free HTG-AP therapeutic methods are in urgent demand. In this regard, natural compounds with potent antioxidative and anti-inflammatory activities represent invaluable resources for development as RA therapeutics.

Resveratrol (3,5,4′-trihydroxy-trans-stilbene, Rev) is a natural polyphenol, which widely exists in various plants, such as *Polygonum cuspidatum*, in fruits, including grapes and berries, in peanuts, and in red wine [9]. Resveratrol plays a regulatory role through a series of mechanisms, including scavenging ROS [10], antioxidative [11], and anti-inflammatory activities [12]. Recent studies have established that resveratrol owns the potential in the prevention or treatment of chronic inflammation-related disorders such as cardiovascular diseases, diabetes, obesity, and cancer [13–16]. Besides, it has an association with some metabolic syndrome diseases including glucose intolerance [17], altered cholesterolemia [18], and hypertriglyceridemia [19]. In view of this, we intended to address the question regarding the effect of resveratrol in HTG-AP and the underlying mechanisms in vivo using a caerulein-induced mice model with a high-fat diet (HFD), for cerulein-induced pancreatitis is the most-well-characterized and widely used experimental model for acute pancreatitis.

Herein, we presented evidence supporting the preventive effect of resveratrol on HTG-AP through alleviating hypertriglyceridemia, oxidative stress, and inflammation in HTG-AP mice and regulating HFD-induced gut microbiota disorders in vivo.

## Methods

### Animals

Forty-eight male C57BL/6 J mice, 18.0–20.0 g, aged 6 weeks, were purchased from Animal Center of West China Medical College, Sichuan University (Chengdu, China). All animals were housed in individual cages under a 12-hour light/dark-cycle environment, provided free water and food, and approved by the ethics committee of Affiliated Hospital of North Sichuan Medical College (No. 2022.03). All efforts were aimed to alleviate animal suffering.

### Materials

Resveratrol (≥99%, Sigma, USA), sodium carboxymethyl cellulose (UNIVAL, Germany), cerulein (Sigma, USA), total cholesterol (TC) and total triglyceride (TG) content assay kits (Solarbio, China), LPS (cusabio, China), MCP-1 (Abcam, USA), TNF-α (Beyotime, China), IL-6 (Abcam, USA) malondialdehyde (MDA) (Solarbio, China), superoxide dismutase (SOD) (Solarbio, China), and total antioxidant capacity (T-AOC) (Solarbio, China) ELISA kits.

### Model preparation and animal grouping

This research was divided into two parts. In the first part of experiments, mice were randomly assigned to 4 groups (*n* = 6) as follows: chow, chow. Rev., HFD, HFD. Rev. Mice were adaptively fed with normal chow or HFD for 2 weeks, and then chow. Rev. and HFD. Rev. groups were intragastric administration with resveratrol (45 mg/kg/d) for 4 weeks. Resveratrol (≥99%, Sigma, USA) was dissolved in 0.5% sodium carboxymethyl cellulose (UNIVAL, Germany). Then all mice were given an intraperitoneal injection of 50 mg/kg sodium pentobarbital and sacrificed by cervical dislocation. Blood and colon tissue were collected and 3 mice from each group were randomly selected to collect colon contents for 16 s rRNA sequencing of gut microbiota.

The second part of experiments was designed to determine the effect of pre-treatment with resveratrol in the HTG-AP mice and the HTG-AP mice model was established according to the reference [20]. In brief, mice were randomly assigned to 4 groups (*n* = 6) as follows: chow + cerulein, chow. Rev. + cerulein, HFD + cerulein, HFD. Rev. + cerulein. The procedures were the same as the first part of experiments. But 24 h after the last intragastric administration with resveratrol, all the groups were intraperitoneal injections of cerulein (Sigma, USA) by two times, each time at a dose of 40 μg/kg body weight. After 12 h, all mice were given an intraperitoneal injection of 50 mg/kg sodium pentobarbital and sacrificed by cervical dislocation. Blood and colon tissue were collected.

### Measurement of TC and TG

Blood biochemical indicators of the lipid profile were assessed. The concentrations of total cholesterol (TC) were measured by Micro total cholesterol (TC) content assay kit (Solarbio, Beijing, China) and triglyceride (TG) was assessed by triglyceride content assay kit (Solarbio, Beijing, China) according to the manufacturer’s instructions.

### Enzyme-linked immunosorbent assay (ELISA)

Blood was collected from mice in each group and centrifuged at 15000 rpm for 10 min to obtain the serum. In the first part of experiments, the levels of proinflammatory cytokines including LPS (cusabio, China), MCP-1 (Abcam, USA), TNF-α (Beyotime, China), and IL-6 (Abcam) were determined by ELISA according to the manufacturer’s instructions. In the second part of experiments, the levels of malondialdehyde (MDA) (Solarbio, Beijing, China), superoxide dismutase (SOD) (Solarbio, Beijing, China), and total antioxidant capacity (T-AOC) (Solarbio, Beijing, China) were measured by ELISA according to the manufacturer’s instructions. The OD value of each well was immediately read at 450 nm.

### Histopathological assessment

Pancreatic tissues from each group were fixed in 4% paraformaldehyde at room temperature, embedded in paraffin, and sectioned at a thickness of 5 μm. In the immumohistochemical staining, TNF-α and MCP-1 antibody (1:400) and related conjugated secondary antibody were used. In the H&E staining, the tissues were stained with hematoxylin and eosin (H&E). The histopathological change was observed under the light microscope (Olympus, Tokyo, Japan) at 400× magnification.

### Western blot assay

After the pancreatic tissues were obtained, proteins were extracted using RIPA lysis buffer (Beyotime, Beijing, China), and protein concentrations were measured using a BCA protein assay kit (Vazyme, Nanjing, China) following the manufacturer’s protocols. The protein samples were then separated by 10% SDS-PAGE and transferred onto polyvinylidene fluoride (PVDF) membranes. Next, the PVDF membranes were blocked by 5% skim milk. After being cultured for 2 h at room temperature, they were then incubated overnight at 4 °C with specific primary antibodies including p65, p-p65, TNF-α, and IL-6. After washing three times, the blots were subsequently incubated with a goat horseradish peroxidase-conjugated secondary antibody for 2 h at room temperature. After four times washing for 10 min in Tris-buffered saline with Tween-20 (TBST), the membranes were detected using a chemiluminescence detection system. The intensity of the bands was quantified by ImageJ software. β-actin served as a loading control.

### DNA extraction

The colon content DNA of mice in each group was extracted with ZR Fecal DNA Extraction Kit (Zymo Research, CA, USA). The buffer solution was added to 200 mg feces from each group to prepare fecal homogenate, and the sediments were centrifuged using a vortex mixer after incubation at 70 °C. Then the supernatant was extracted, and inhibitors were added. The sediments were centrifuged, aspirated the supernatant again. The buffer solution was added and incubated at 70 °C for 10 min, and 200 μl absolute ethanol was added ultimately. After the sample was purified, the DNA sample is obtained, which is quantified by an ultraviolet spectrophotometer and tested for purity. After this, the DNA quality is analyzed by agarose gel electrophoresis.

### 16sRNA sequencing of gut microbiota

Bacterial RNA was amplified by RT-PCR targeting the V3-V4 hypervariable regions of the 16 s RNA gene and using specific primers (319F: 5′ ACTCCTACGGGAGGCAGCAG 3′; 806R: 3′ ACTCCTACGGGAGGCAGCAG 5′). Amplicons were pooled and paired-end sequenced on an Illumina MiSeq (Illumina) in the Shanghai Personal Biotechnology Co., Ltd. (Shanghai, China). The Quantitative Insights Into Microbial Ecology (QIIME, v1.8.0) pipeline was employed to process the sequencing data, as previously described. Sequence processing and microbial composition analysis were performed with the Quantitative Insights into Microbial Ecology (QIIME) software package, version 1.9.1. After quality filters, the remaining high-quality sequences were clustered into operational taxonomic units (OTUs) at 97% sequence using the reference-based USEARCH (version 5.2) pipeline in QIIME, using the May 2013 release of the GreenGenes 99% OTU database as a closed reference. The raw data and sequencing sample information have been submitted to the SILVA database to classify.

### Statistical analysis

All data are expressed as mean ± SD. Statistical analysis was performed using GraphPad Prism 7 (GraphPad software, USA). Statistical differences among the groups were determined using one-way ANOVA or two-way ANOVA to compare differences between experimental and control groups. Results with *p* < 0.05 were considered statistically significant. All experiments were performed at least in triplicate.

## Results

### Resveratrol decreased serum levels of TC and TG in HFD and HTG-AP mice

**Fig. 1:**
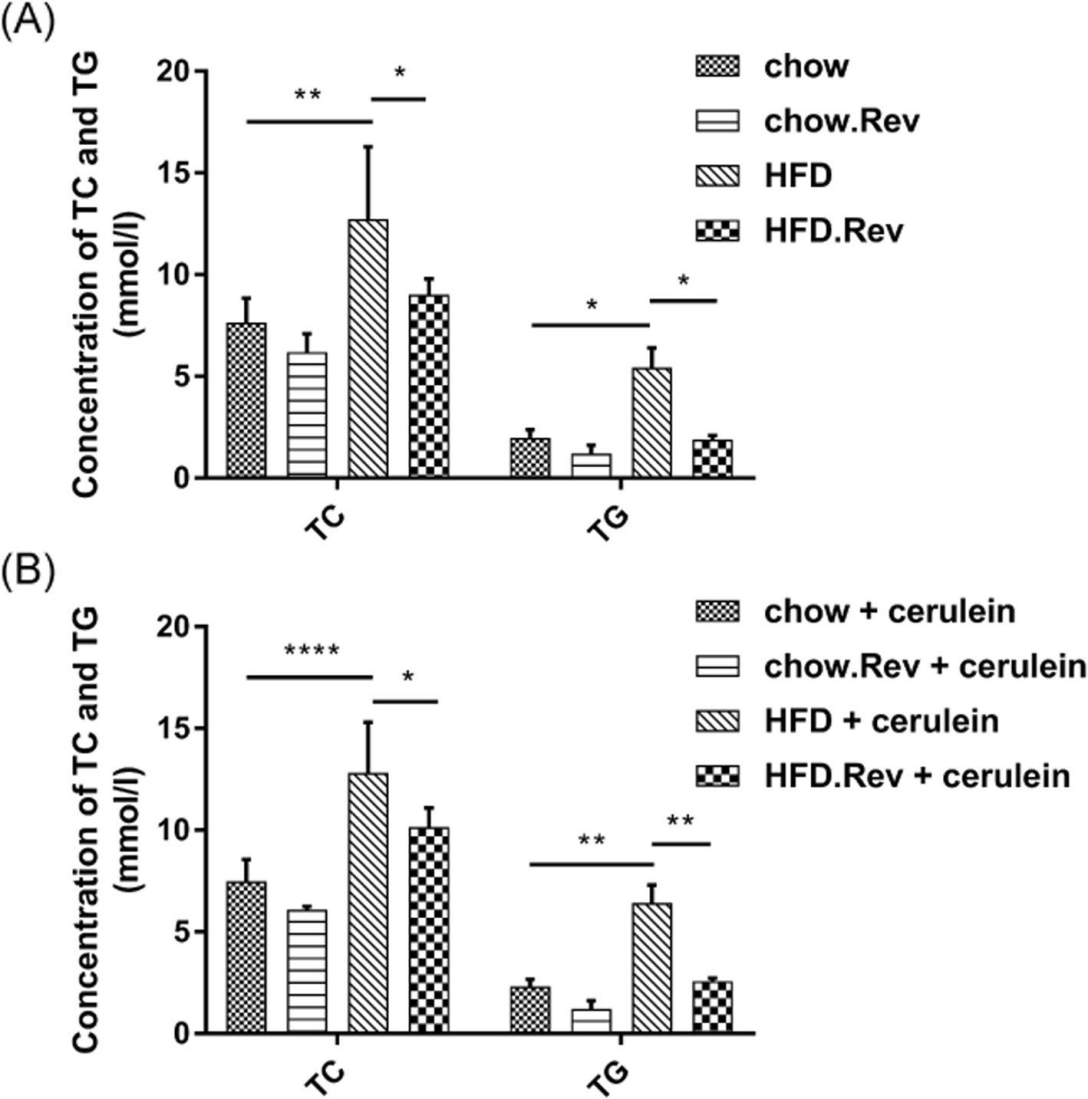
Serum lipid profiles after treatment with resveratrol in different models. The concentrations of TC and TG in HFD model (**A**), TC and TG in HFD + cerulein model (**B**) were analyzed among different groups. TC, total cholesterol; TG, triglyceride. Bars represent the mean ± S.D. from three independent experiments. *p < 0.05, **p < 0.01, ****p < 0.0001

The levels of serum TC and TG were conducted to evaluate the alterations in lipid profiles in different groups. As shown in Fig. 1, the serum concentration of TC and TG varied among groups. In the HFD model group, the TC and TG levels were markedly higher than those in the chow group, which indicated that high lipid levels were associated with HFD (Fig. 1A). Similarly, the levels of TC and TG in the HFD + cerulein model group (HTG-AP) were significantly higher than those in the chow + cerulein group (Fig. 1B). However, administration of resveratrol markedly decreased the TC and TG levels compared with the HFD model group and HFD + cerulein model group in the same way.

### Resveratrol alleviated histopathological damage of pancreatic tissue in HFD and HTG-AP mice

**Fig. 2:**
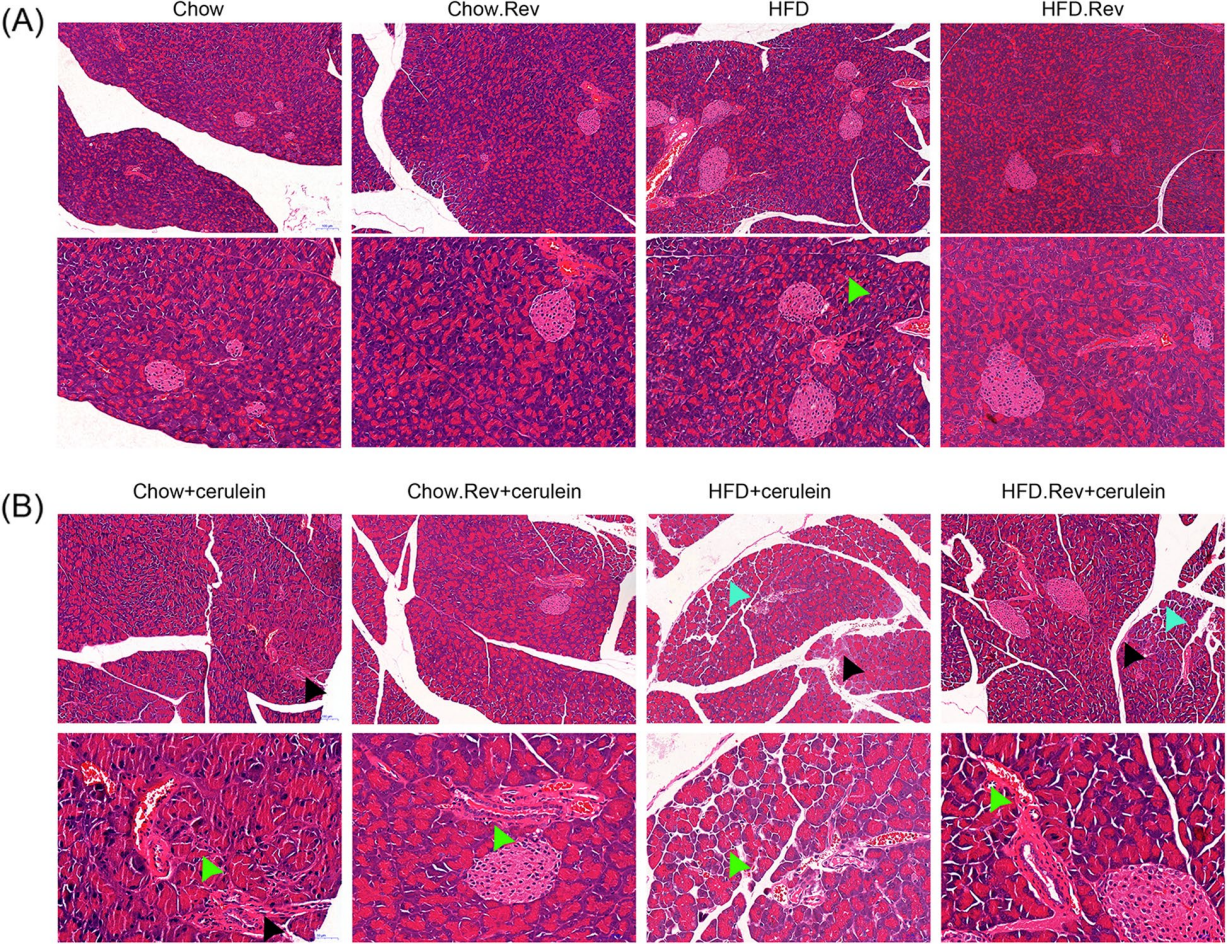
Pathological changes in pancreatic tissues following treatment with resveratrol. Pancreatic tissues stained with H&E were visualized with a light microscope at a magnification of × 200 and × 400. Scale bar = 100 μm. **A** Pathological changes in HFD mice; **B** Pathological changes in HTG-AP mice. Green arrow: lymphocytic infiltration, blue arrow: acinar separation, and black arrow: acinar epithelial cells are denaturated and necrotic

The pancreatic tissues from all the groups were collected and stained with H&E to observe the histopathological change. In the HFD model group, the pancreatic tissue of chow group displayed a clear tissue structure with no obvious abnormality in the pancreatic ducts, islet, and acini. No obvious inflammatory cell infiltration was observed. And the histopathological characteristics in chow. Rev. group was similar to the chow group. However, pancreatic tissue in the HFD group displayed interstitial edema, and a small number of inflammatory cells infiltrated into the perivascular and stroma. These cells were mainly lymphocytes with round nuclei and deep staining. Nevertheless, an improvement in pathological changes was observed following resveratrol treatment compared with those of the HFD group (Fig. 2A). Likewise, in the HTG-AP model group, pancreatic tissue of chow + cerulein group displayed local acinar epithelial cell degeneration and necrosis, nucleus contraction, a small number of inflammatory cells infiltrated in the stroma and around the blood vessels, and these cells were mainly round hyperchromatic lymphocytes. And the pathological changes in HFD + cerulein group had similarities to those of chow + cerulein group following the hyperplasia of acinar stromal and slight separation of some acinus. Encouragingly, all these pathological changes can be reversed by treatment with resveratrol (Fig. 2B).

### Resveratrol down-regulated the expressions of MCP-1 and TNF-α in HFD and HTG-AP mice

**Fig. 3:**
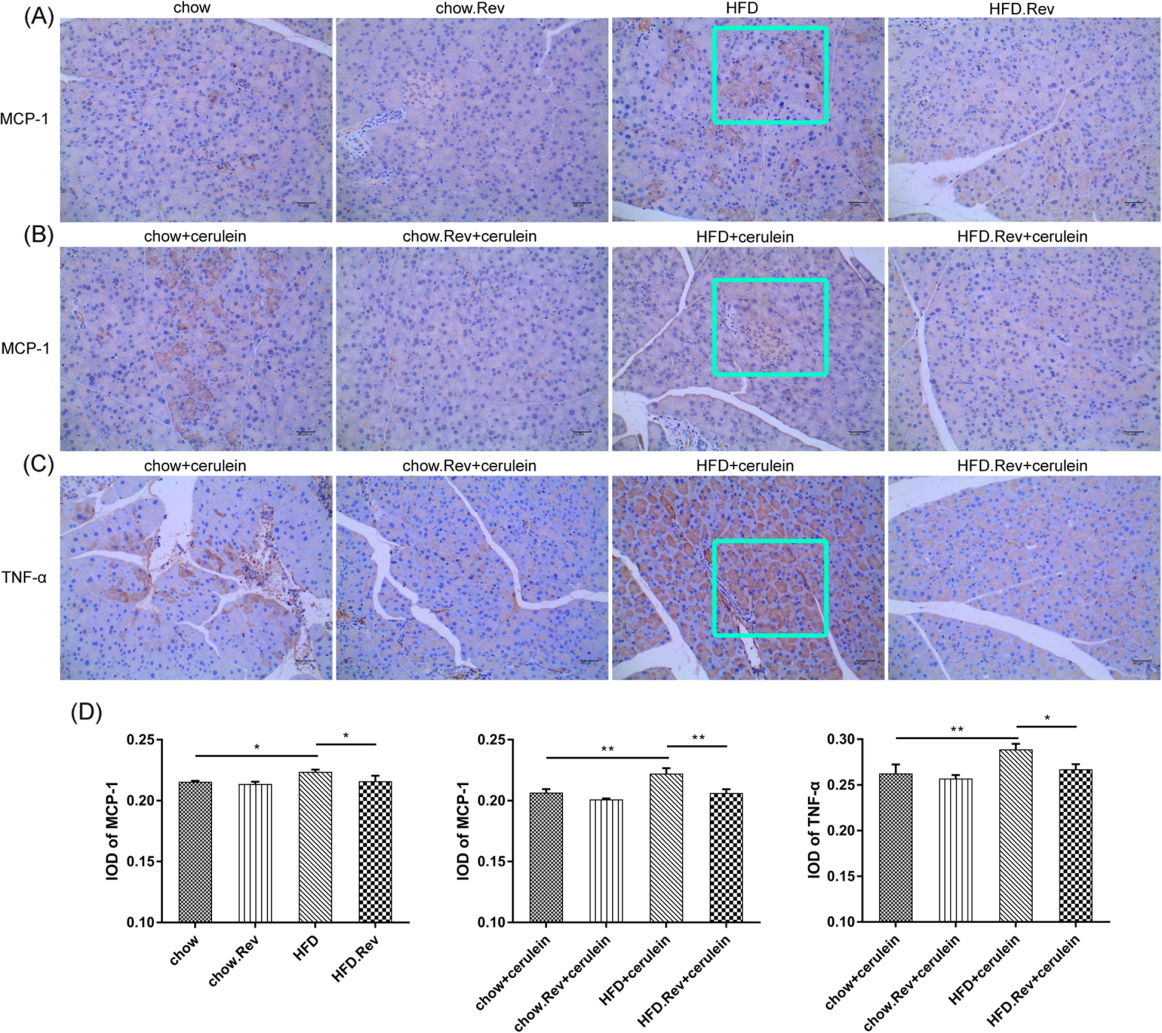
Immunohistochemical staining of HFD and HTG-AP model mice for MCP-1 and TNF-α. **A** The expression of MCP-1 in HFD model group. Resveratrol-treated mice showed lower level of MCP-1 than the model group. **B** The expression of MCP-1 in HTG-AP model. **C** The expression of TNF-α in HTG-AP model group. **D** Bar graphs of the integral optical density (IOD) of tissue MCP-1 and TNF-α levels. Blue box: The target protein is deposited in the pancreas tissue *p < 0.05, **p < 0.01

Researchers have demonstrated that MCP-1 and TNF-α were overexpressed when the pancreatic tissue was damaged [21, 22]. Therefore, to evaluate the alterations in different groups, the expression of MCP-1 was measured in the HFD model group. The expression of MCP-1 and TNF-α were conducted in the HTG-AP model group. As for the expression of MCP-1, it was over-expressed in the HFD-treated, cerulein-treated, and HFD + cerulein-treated group (Fig. 3A and B) and this can be reversed by adding resveratrol. Similarly, as for the expression of TNF-α, it was over-expressed in the cerulein-treated and HFD + cerulein-treated group (Fig. 3C) compared with the resveratrol-treated group. Furthermore, the integral optical density (IOD) values of MCP-1 and TNF-α were shown. The expressions of MCP-1 and TNF-α were both up-regulated in HFD or HFD + cerulein group, but the addition of resveratrol can revert this (Fig. 3D).

### Resveratrol reduced serum levels of inflammatory cytokines in HFD mice

**Fig. 4:**
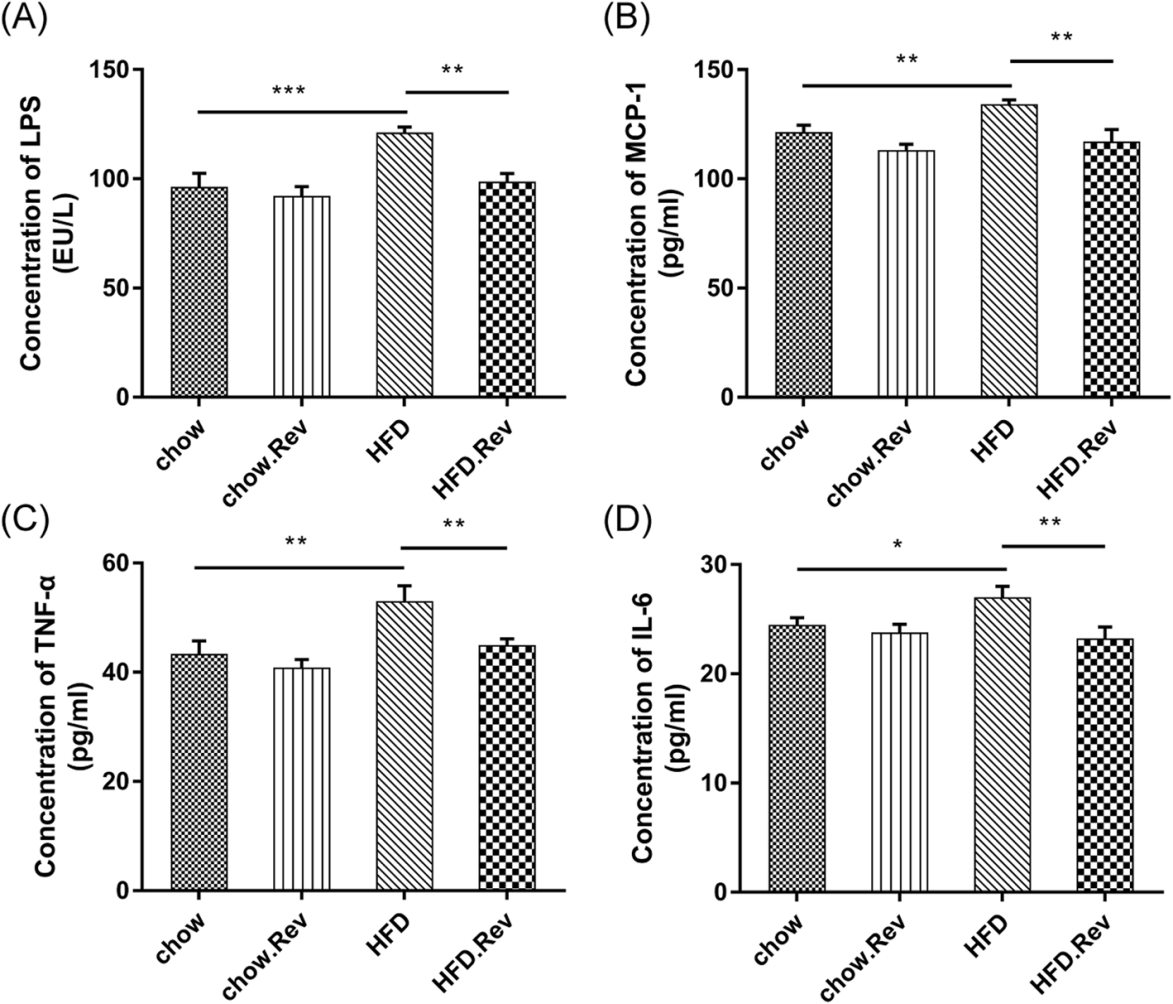
Serum proinflammatory cytokines levels, including LPS, MCP-1, TNF-α and IL-6. The concentrations of (**A**) LPS, (**B**) MCP-1, (**C**) TNF-α and (**D**) IL-6 in the different groups were determined by ELISA. Bars represent the mean ± S.D. from three independent experiments. *p < 0.05, **p < 0.01, ***p < 0.001

The expression levels of inflammatory cytokines, including LPS, MCP-1, TNF-α, and IL-6 in the serum of HFD mice were measured by ELISA. The higher levels of LPS, MCP-1, TNF-α, and IL-6 in the HFD group compared with those in the chow group suggested that HFD may be related to the overexpression of inflammatory cytokines and the inflammatory response (Fig. 4). The HFD group displayed the highest levels of these cytokines compared with the chow group. However, the levels of LPS (Fig. 4A), MCP-1 (Fig. 4B), TNF-α (Fig. 4C), and IL-6 (Fig. 4D) were all significantly reduced following the administration of resveratrol. These results suggested that resveratrol might have a therapeutic effect against inflammation in HFD.

### Resveratrol decreased oxidative stress level in HTG-AP mice

**Fig. 5:**
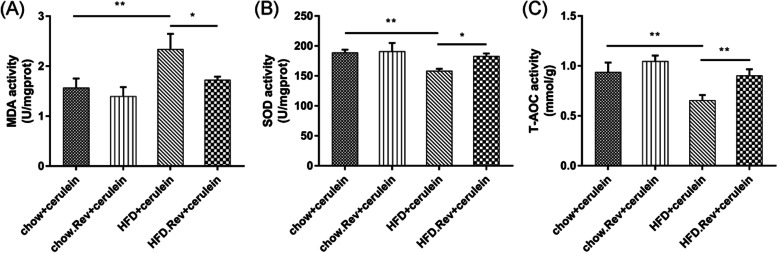
The levels of oxidative stress-related markers in pancreatic tissue. The activities of (**A**) MDA, (**B**) SOD, and (**C**) T-AOC in the different groups were determined by ELISA. Bars represent the mean ± S.D. from three independent experiments. *p < 0.05, **p < 0.01

The oxidative stress-related markers in pancreatic tissue were measured to determine the antioxidant effects of resveratrol. The MDA activity, standing for lipid peroxidation, was markedly higher in HFD + cerulein group compared with the chow + cerulein, and it was reduced following resveratrol treatment (Fig. 5A). Additionally, the SOD activity, represent for free radical level, was significantly lower in HFD + cerulein group. The resveratrol-treated group suffered an increase in the activity of SOD (Fig. 5B). Ultimately, T-AOC activity, standing for total antioxidant level, in the resveratrol-treated group was higher than HFD + cerulein group (Fig. 5C). These results manifested that resveratrol can reverse the oxidative stress caused by cerulein.

### Resveratrol restrained NF-κB signaling pathway in HTG-AP mice pancreatic tissues

**Fig. 6:**
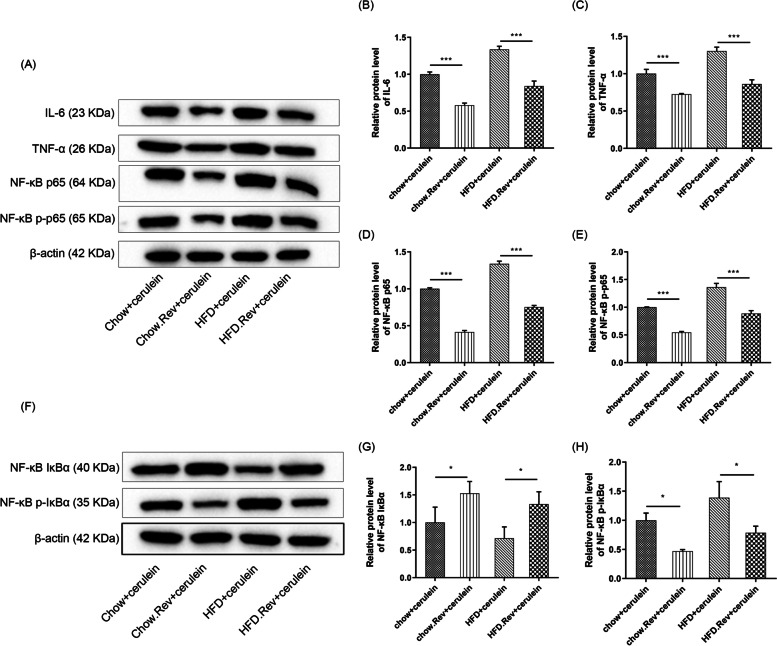
Effect of resveratrol on the NF-κB signaling pathway. **A** The protein levels of IL-6, TNF-α, NF-κB p65 and p-p65 were measured by western blotting. **B** Relative protein level of IL-6; **C** Relative protein level of TNF-α; **D** Relative protein level of NF-κB p65; **E** Relative protein level of p-p65. **F** The protein levels of NF-κB IκBα and p-IκBα and p-p65 were measured by western blotting. **G** Relative protein level of NF-κB IκBα; **H** Relative protein level of NF-κB p-IκBα. β-actin served as a loading control. Bars represent the mean ± S.D. from three independent experiments. *p < 0.05, **p < 0.01

It is reported that the activation of nuclear factor-κB (NF-κB) signaling pathway can induce the expression and release of its downstream inflammatory cytokine IL-6 [23]. And TNF-α can serve as an activator of the NF-κB pathway [24]. The results of WB analysis for IL-6, TNF-α, and NF-κB pathway-related proteins in pancreatic tissue of HTG-AP mice were shown in Fig. 6. Bands of IL-6, TNF-α, NF-κB p65, phosphorylation-p65 (p-p65), IκBα and p-IκBα were displayed (Fig. 6A&H). The expressions of IL-6 (Fig. 6B), TNF-α (Fig. 6C), NF-κB p65 (Fig. 6D), p-p65 (Fig. 6E), and p-IκBα (Fig. 6H) were all significantly decreased in both chow. Rev. + cerulein and HFD. Rev. + cerulein groups, and similar results were shown in chow groups. These results suggested that resveratrol inhibited the activation of the NF-κB signaling pathway in pancreatic tissues, thus suppressing the activation of pro-inflammatory signaling, concerning the expressions of IL-6 and TNF-α.

### Resveratrol influenced the microbial diversity and the structure of community of feces in HFD mice

**Fig. 7:**
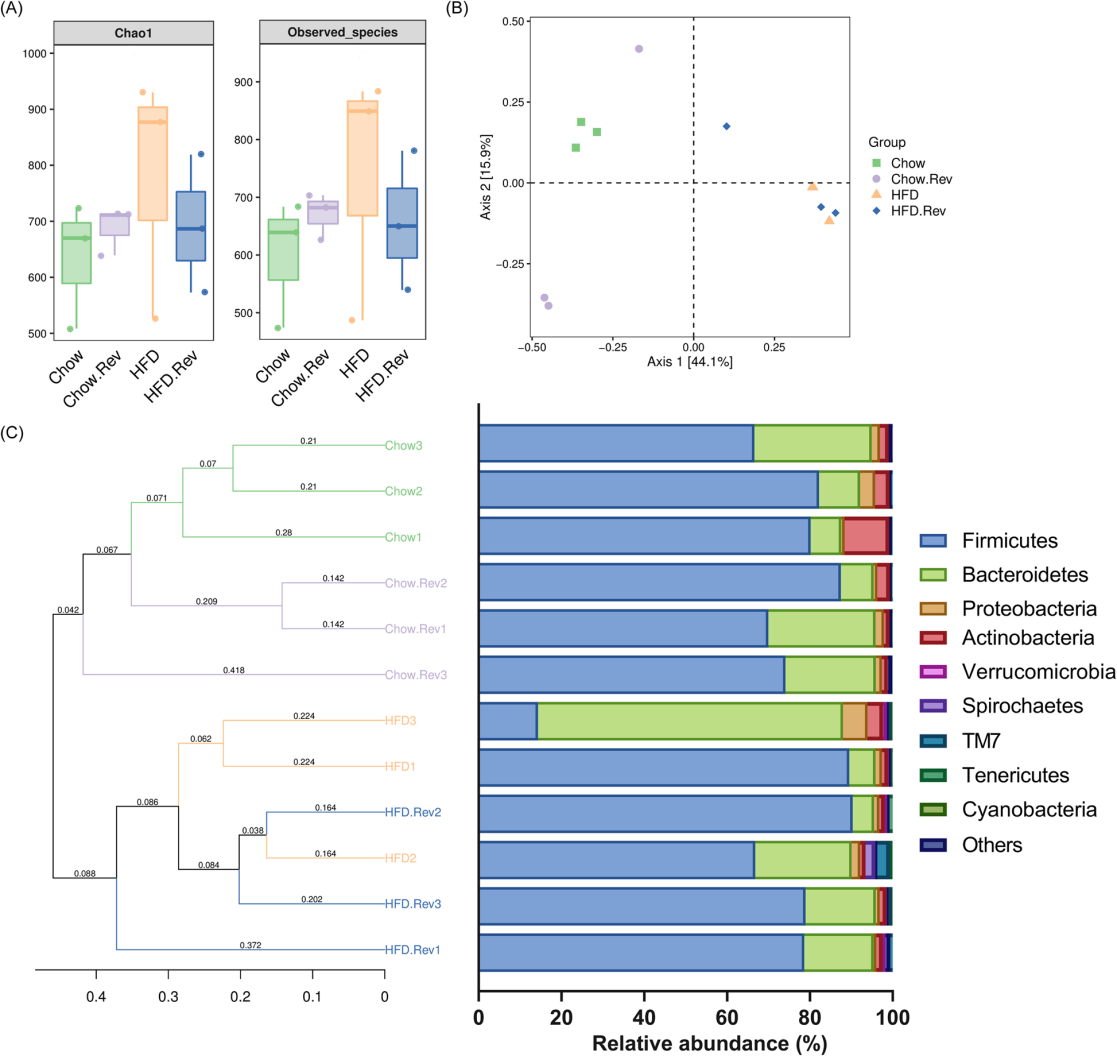
Effect of resveratrol on microbial diversity and structure of community of feces in HFD mice. **A** Richness and diversity of fecal microbiota. No significant difference was observed among groups. **B** Principal coordinate (PCoA) analysis of Bray-Curtis distance. **C** Cluster analysis of unweighted pair group method with arithmetic mean (UPGMA) based on Bray-Curtis distance

To explore the effect of resveratrol on gut microbiota in HFD mice, fecal samples were collected to analyze its diversity and richness. Multiple alpha diversity metrics of richness and diversity revealed that no significant difference was observed between groups, as shown in Chao1 and the observed species method (Fig. 7A). Bray-Curtis distance-based PCoA analysis was employed to determine the similarities and differences in the composition of gut microbiota among groups. HFD group showed a difference in gut microbiota compared with the chow group. HFD + Rev. group showed a movement in the first principal component (PC1) towards the direction of the chow group, thus HFD + Rev. shortened the distance with the chow group (Fig. 7B). Hierarchical clustering analysis revealed that the microbial communities in the resveratrol-treated group showed more similarities to those in the chow group (Fig. 7C). In short, resveratrol treatment can reverse the HFD-induced variations.

### Resveratrol affected the composition of gut microbiota in HFD mice

**Fig. 8:**
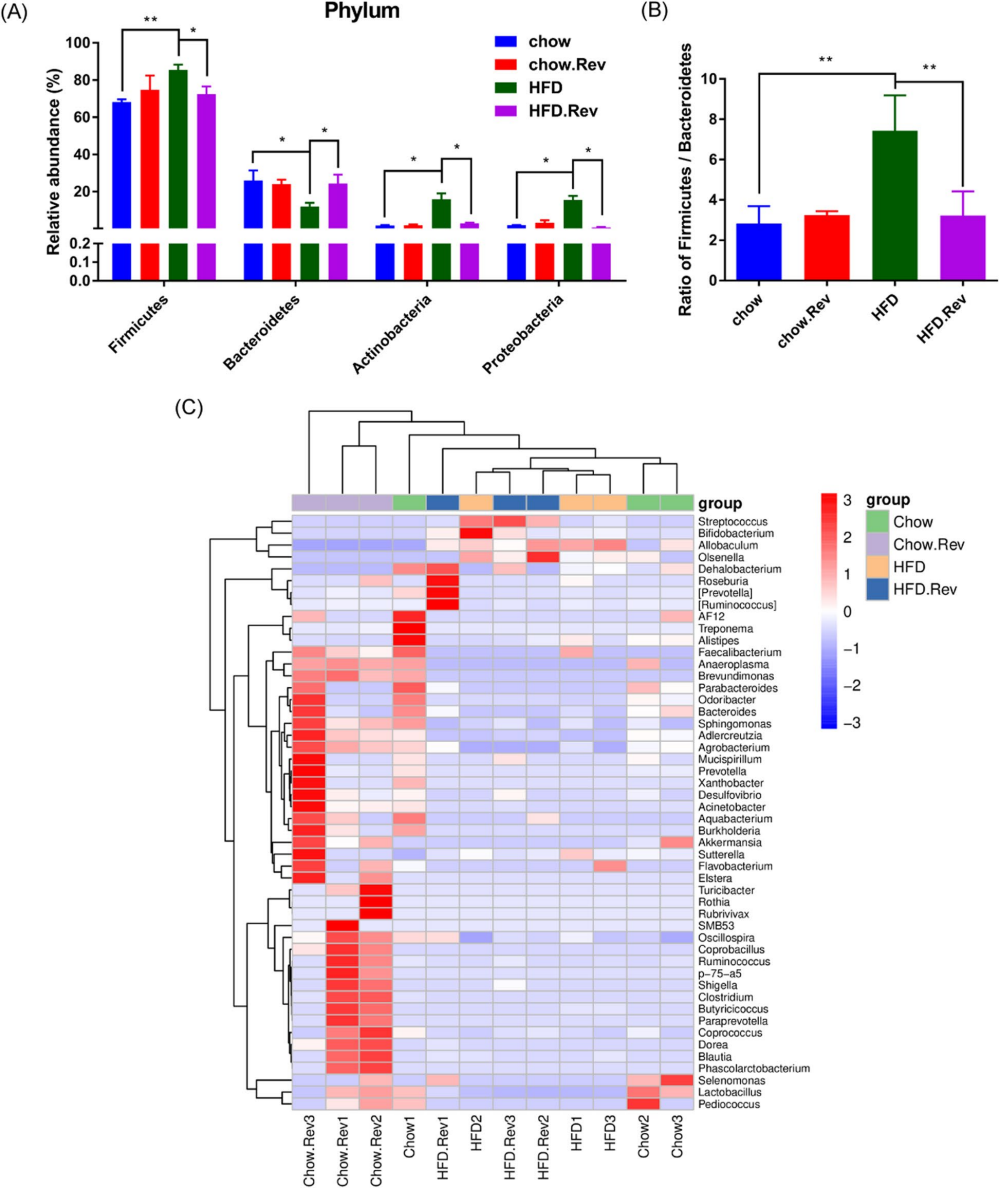
Effect of resveratrol on composition of gut microbiota in HFD mice. **A** Difference in the relative abundance of *Firmicutes*, *Bacteroidetes*, *Actinobacteria* and *Proteobacteria* phylum among groups. **B** The ratio of *Firmicutes/Bacteroidetes* in phylum level. **C** The heat map of top 50 abundant genus. Double hierarchical dendrogram shows the bacterial distribution. *p < 0.05, **p < 0.01

**Fig. 9:**
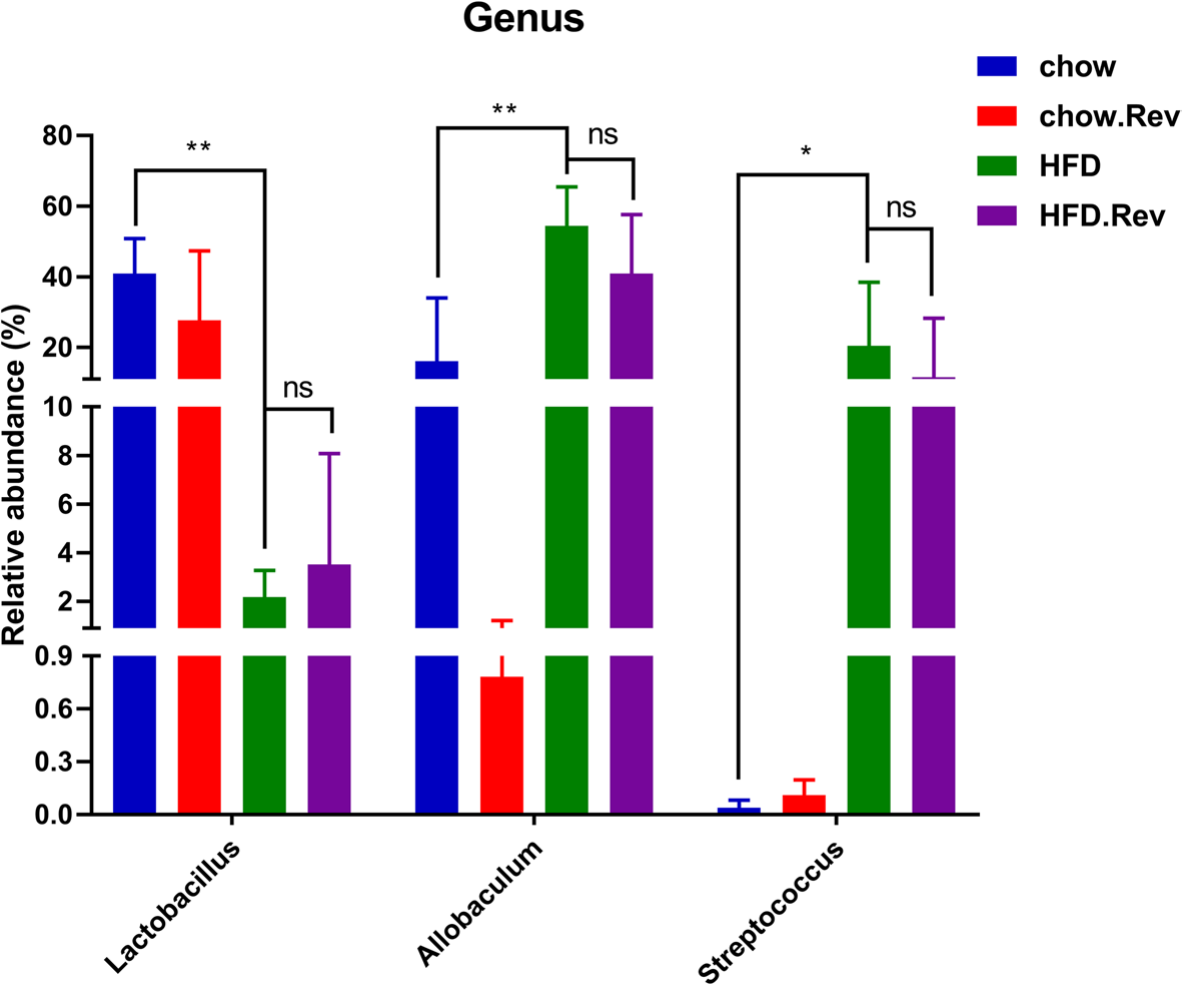
Resveratrol-related alterations at genus levels of *Allobaculum*, *Lactobacillus* and *Streptococcus*. Bars represent the mean ± S.D. from three independent experiments. *p < 0.05, **p < 0.01

The composition of gut microbiota was shown in Fig. 8A. In general, HFD-relative changes in fecal gut microbiota were characterized with a significantly higher relative abundance of *Firmicutes*, *Actinobacteria*, and *Proteobacteria* but a markedly lower relative abundance of *Bacteroidetes*. After treatment with resveratrol, it decreased the relative abundance of *Firmicutes*, *Proteobacteria*, and *Actinobacteria*, and increased the relative abundance of *Bacteroidetes*. Furthermore, the ratio of *Firmicutes/Bacteroidetes* was increased highly in the HFD group compared with the chow group and resveratrol treatment can reverse this (Fig. 8B). Consistent with beta diversity, clustering analysis of the top 50 genera highlighted differences in their distributions after treatment with resveratrol (Fig. 8C). Then, we analyzed the difference in the genus of each group and the statistically significant genera were shown. Compared with the chow group, the HFD mice showed a higher level in the relative abundance of *Allobaculum* and *Streptococcus* but a lower level in the relative abundance of *Lactobacillus* (Fig. 9). Specifically, compared with the HFD group, the HFD. Rev. group promoted the recovery of the relative abundance of *Allobaculum* and *Lactobacillus* and decrease the relative abundance of *Streptococcus*. In addition, in the chow and chow. Rev. groups, we found that the relative abundance of these three genera was consistent with the HFD and HFD. Rev. groups (Fig. 9).

## Discussion

Acute pancreatitis (AP) caused by hypertriglyceridemia (HTG-AP) is usually associated with a repeated attack of AP [25]. HTG can be divided into primary and secondary types. Primary HTG is caused by a high-fat, high-carbohydrate diet and other genetic and environmental factors, as well as lack of physical activity which can lead to disorders of TG synthesis and metabolism [26]. Secondary HTG is usually induced by unrecognized diseases, including obesity, diabetes, pregnancy, metabolic syndrome, and drugs such as estrogen and tamoxifen can also lead to the occurrence of HTG [6]. Studies have shown that the pathogenesis of HTG-AP is related to the inflammatory response, [27] microcirculatory disorder, [28] Ca^2+^ overload and endoplasmic reticulum stress, [29, 30] oxidative stress [31] and accumulation of free fatty acid [32]. A retrospective analysis showed that HTG-AP patients are generally younger than AP, and are more likely to suffer from cardiopulmonary and renal insufficiency and systemic inflammatory response syndrome (SIRS) [7]. There are some effective treatments on HTG-AP, such as insulin, heparin, plasmapheresis, and anti-HTG drugs. The chemopreventive effects of resveratrol in a rat model of cerulein-induced AP have been reported [33], however, whether resveratrol has a protective effect on HTG-AP is unknown. In our study, we successfully established the HTG-AP model according to the reference [20], finding that HTG-AP was more serious than AP, and proved that the resveratrol pre-treatment had a preventive effect on HTG-AP.

NF-κB transcription factor plays an important role in inflammation, immune response, survival and apoptosis [34]. This pathway regulates the production of pro-inflammatory cytokines, the aggregation of inflammatory cells, and the promotion of inflammatory response. Many studies have shown that the NF-κB pathway is involved in the inflammatory process and cancer development. For example, Th17 type cytokines, IL-6 and TNF-α can synergistically activate STAT3 and NF-κB pathways to promote the growth of colorectal cancer cells [35]. In addition, STAT3 and NF-κB pathways are also active in pancreatic cancer [36]. Clinical evidence shows that NF-κB pathway components play an important role in tumorigenesis and development, regulating gene expression related to cell survival and proliferation, drug resistance, metastasis, and angiogenesis [37]. Therefore, NF-κB can be used as a molecular target for some cancers.

Nowadays, several studies have been identified that some phytochemicals have inhibitory effects on the NF-κB pathway [38]. Among the polyphenols, resveratrol, curcumin, epigallocatechin gallate, genistein and cardamom have been the most well-studied. They have the ability to block NF-κB nuclear transport or restrain NF-κB activation to inhibit the proliferation ability of cancer cells. For example, resveratrol can treat glioblastoma multiforme by inhibiting PI3K/Akt/NF-κB signal transduction and inhibiting MMP-2 expression [39]; curcumin has a therapeutic effect on oxaliplatin-resistant colon cancer cell lines by inhibiting CXC chemokine/NF-κB signaling pathway [40]; Epigallocatechin gallate (EGCG) can treat nasopharyngeal carcinoma via regulating the cellular localization of NF-κB p65 and reducing the transcriptional regulation effect of NF-κB p65 on Twist1 expression [41].

Gut microbiota is closely associated with lipid metabolism disorder and systemic inflammation of obese mice [42]. Currently, *Firmicutes*, *Bacteroides*, *Actinobacteria* and *Proteobacteria* accounted for more than 90% of the gut microbiota [43]. Studies have shown that long-term HFD changed the gut microbiota, leading to increased intestinal permeability, mucosal immune response, obesity and chronic inflammation [44]. The prevalence of *Firmicutes*, *Actinobacteria* and *Proteobacteria* is positively associated with HFD, whereas *Bacteroides* show the opposite effect. Obesity and obesity-related pathologies are related to the occurrence of chronic low-grade inflammation [45]. Most patients with obesity exhibit increased circulating levels of inflammatory markers such as IL-6, IL-1, TNF and MCP1 [46]. As we know, some gut microbiota plays a role in the pro-inflammatory effect and others have an anti-inflammatory effect. *Lactobacillus* is a well-known probiotic that is proven to be related to reducing colitis in several models of inflammatory bowel diseases [47]. But the prevalence of *Allobaculum* level has been shown to be associated with neuronal and intestinal inflammation [48]. *Streptococcus pneumonia* is the most common type of *streptococcus*, and it can cause diseases such as pneumonia, meningitis and otitis media [49]. It has been reported in the HFD mice that the improvement of resveratrol on gut microbiota [50]. Indeed, the resveratrol-treated HFD group can up-regulate the relative abundance of anti-inflammatory *Lactobacillus* but down-regulate the relative abundance of pro-inflammatory *Allobaculum* and *streptococcus* compared with the HFD group. Similar results were observed in the chow and chow. Rev. group. These results demonstrated that resveratrol pre-treatment improved the gut microbiota in the HFD mice, thereby reducing the pancreatic damage following induced by caerulein.

In this study, the HFD mice model and HFD + cerulein (HTG-AP) mice model were established to measure the effect of resveratrol. In the HFD mice model, the expression levels of inflammatory and chemotactic cytokines TNF-α, LPS, IL-6, MCP-1 and the damage of pancreatic tissue were decreased, and the composition of gut microbiota was different. Also, in the HTG-AP mice model, resveratrol pre-treatment decreased the expressions of TNF-α, MCP-1, MDA the injury of pancreatic tissue, increased the levels of SOD and T-AOC. The results indicated that resveratrol pre-treatment can inhibit the secretion of pro-inflammatory cytokines and promote the antioxidant stress capacity. Furthermore, the NF-κB signaling pathway was inhibited by resveratrol, and the expressions of TNF-α and IL-6 related to the NF-κB signaling pathway activation were also decreased. All these results suggested that resveratrol pre-treatment can reduce oxidative stress, attenuate pancreatic tissue injury and restrain inflammatory response by inhibiting the NF-κB signaling pathway.

## Conclusion

Using in vivo experiments, we provided evidence that HTG could promote AP injury, aggravating inflammation and oxidative stress. Moreover, the resveratrol pre-treatment could attenuate pancreas injury, inflammation, and oxidative stress in the HTG-AP mice, via restraining the NF-κB signaling pathway and regulating gut microbiota. Our study proved that the short-term resveratrol pre-treatment had a preventive effect on HTG-AP. However, the effects of long-term resveratrol pre-treatment and treatment on HTG-AP need more research.

## Data Availability

The initial data used to support the findings of this study are available from the corresponding author upon request. The sequencing data used in this study are stored in NCBI SRA database (SUB10626305). https://www.ncbi.nlm.nih.gov/sra/PRJNA778434.
